# Rationale, design and methodology for Intraventricular Pressure Gradients Study: a novel approach for ventricular filling assessment in normal and falling hearts

**DOI:** 10.1186/1749-8090-6-67

**Published:** 2011-05-10

**Authors:** Miguel Guerra, Mário J Amorim, João C Mota, Luís Vouga, Adelino Leite-Moreira#

**Affiliations:** 1 Faculty of Medicine of University of Oporto, Department of Physiology, Alameda Professor Hernâni Monteiro, 4202-451 Porto, Portugal; 2 Centro Hospitalar de Vila Nova de Gaia/Espinho, EPE, Department of Cardiothoracic Surgery, Rua Conceição Fernandes, 4434-502 Vila Nova de Gaia, Portugal; 3 Hospital de São João, Department of Cardiothoracic Surgery, Alameda Professor Hernâni Monteiro, 4202-451 Porto, Portugal

## Abstract

**Background:**

Intraventricular pressure gradients have been described between the base and the apex of the left ventricle during early diastolic ventricular filling, as well as, their increase after systolic and diastolic function improvement. Although, systolic gradients have also been observed, data are lacking on their magnitude and modulation during cardiac dysfunction. Furthermore, we know that segmental dysfunction interferes with the normal sequence of regional contraction and might be expected to alter the physiological intraventricular pressure gradients. The study hypothesis is that systolic and diastolic gradients, a marker of normal left ventricular function, may be related to physiological asynchrony between basal and apical myocardial segments and they can be attenuated, lost entirely, or even reversed when ventricular filling/emptying is impaired by regional acute ischemia or severe aortic stenosis.

**Methods/Design:**

*Animal Studies: *Six rabbits will be completely instrumented to measuring apex to outflow-tract pressure gradient and apical and basal myocardial segments lengthening changes at basal, afterloaded and ischemic conditions. Afterload increase will be performed by abruptly narrowing or occluding the ascending aorta during the diastole and myocardial ischemia will be induced by left coronary artery ligation, after the first diagonal branch.

*Patient Studies: *Patients between 65-80 years old (n = 12), both genders, with severe aortic stenosis referred for aortic valve replacement will be selected as eligible subjects. A high-fidelity pressure-volume catheter will be positioned through the ascending aorta across the aortic valve to measure apical and outflow-tract pressure before and after aortic valve replacement with a bioprosthesis. Peak and average intraventricular pressure gradients will be recorded as apical minus outflow-tract pressure and calculated during all diastolic and systolic phases of cardiac cycle.

**Discussion:**

We expect to validate the application of our method to obtain intraventricular pressure gradients in animals and patients and to promote a methodology to better understand the ventricular relaxation and filling and their correlation with systolic function.

## Background

Normal diastolic function of the left ventricle (LV) can be defined as the ability of the ventricle to adequately fill under low filling pressures. The hallmark of diastolic dysfunction is the impaired capacity to fill or maintain stroke volume without a compensatory increase in filling pressures [1,2]. Study of diastolic LV function should primarily be inspired by the impact that diastolic dysfunction has on symptoms and prognosis. Actually, diastolic dysfunction is present in a number of cardiac diseases and often precedes LV systolic dysfunction, leading to symptoms of heart failure in patients with preserved systolic function [3].

As early as 1930, Katz [4] already speculated that diastole was not entirely a passive process and the LV had the ability to "exert a sucking action to draw blood into its chamber." But it was only in 1979 that Ling et al. [5] first described, in a canine model, intraventricular pressure gradients (IVPG) during relaxation and filling of the LV. In 1988, Courtois et al. [6] observed, also in a canine model, a significant sub-basal-apical early diastolic pressure gradient along the LV inflow tract with minimum pressure in the apex speculating suction of the blood toward the LV apex. When subsequently it was shown that these gradients were diminished by ischemia and related to systolic function [7], the concept that they reflected recoil was born. Moreover, when Nikolic et al. [8] in 1995 demonstrated IVPG during early diastole in filling as well as in non-filling heart beats, the hope that IVPG would become an index for isovolumic and early ventricular relaxation was substantiated.

Therefore to describe LV diastolic function comprehensively, it is crucial the precise characterization of the transmitral and intraventricular pressure-flow relation. Early diastole is not amenable to analysis with simple passive-filling models, and any complete description of diastole must account for ventricular suction and for the presence of regional pressure oscillations which play an important role in normal ventricular filling [9]. In fact, the observation that the apical region fills first and begins to oscillate while filling is still occurring in the basal region is consistent with a model of diastolic function in which it can be inferred that suction is completed earlier near the apex than near the base [6,7]. Later [10], it was also demonstrated that in both animals and humans the pressure gradient between the ventricular apex and outflow tract strongly correlated with peak early transmitral flow and stroke volume and markedly increased during volume loading and decreased during reduced LV filling by caval constriction. Furthermore, in 2001, Firstenberg et al. [11] confirmed the existence of IVPG during early diastolic filling in humans and demonstrated that improvements in LV systolic and diastolic function, through surgical myocardial revascularization and/or LV remodeling, result in increases in IVPG. In fact, the same group has shown in patients with hypertrophic cardiomyopathy that diastolic IVPG are lower than in healthy subjects and improve after percutaneous septal ablation [12]. Although systolic IVPG has been also observed between the LV apex and the subaortic area [10], data are lacking on the magnitude of these gradients and its modulation during systolic and diastolic function impairment. Actually, regional ischemia interferes with the normal sequence of regional contraction and might be expected to alter the physiological diastolic and systolic IVPG.

These observations suggest the critical importance of IVPG to ensure efficient LV diastolic filling and allow us hypothesizing that any condition which interferes with the normal sequence of regional relaxation might be expected to change the physiological IVPG pattern. Actually, several studies have demonstrated that LV function is nonuniform in healthy hearts [13,14]. Peak shortening is larger in the lateral wall than in the septum and increases from the base to apex [15]. Besides variations in the degree of shortening, variations in the timing of shortening have been reported including early onset and late peak of shortening in the lateral wall [16]. Moreover, mechanical interaction between different myocardial segments has been studied extensively during regional ischemia, a condition in which regional myocardial function of the ischemic segment is decreased but that of the adjacent normal myocardium may be increased [17,18]. Understanding the origin of normal regional differences in LV myocardial function may give insight in pathological nonuniformities.

A variety of other disorders are associated with diastolic dysfunction, such as hypertrophy, structural alterations of the myocardium with increased fibrosis, myocardial scarring, or infiltrative processes [19]. In addition to these changes, physiological abnormalities of the LV with impaired relaxation, decreased diastolic filling, and increased stiffness of the myocardium can be observed [20]. In patients with aortic stenosis (AS), the most common cause for diastolic dysfunction is LV hypertrophy [21,22]. In subjects with asymptomatic severe AS, increased LV mass index was found to be an independent predictor for the development of symptoms [23]. Although it has been previously shown that aortic valve replacement (AVR) may lead to immediate hemodynamic improvement and to prolongation of survival [24,25], it has been reported that regression of myocardial hypertrophy after relief of the hemodynamic burden is a process that may continue for decades after AVR [26]. However, abnormal exercise hemodynamics may persist late after AVR despite a normal systolic response [27], suggesting impaired diastolic function in these patients which may be revealed by acute and early IVPG alteration.

Despite the apparent importance of IVPG in diastolic function evaluation, they have never been utilized in clinical cardiology, due to the complexity of their acquisition. Whereas regional pressure differences between the LV, the LV outflow tract, and the aorta during ejection have been recognized for some time [28], the importance of regional pressure differences within the ventricle during diastole and systole has only recently gained attention. Moreover, LV dysfunction may be underestimated when only LV ejection fraction is evaluated. Actually, tissue Doppler imaging [29] and 2-dimensional strain [30] analysis of longitudinal myocardial function have shown to be superior in detecting subtle deteriorations of contractility. However, methodology to provide means for earlier diagnosis of global or regional myocardial disease remains an issue of study and necessary research [31-33].

In conclusion, we hypothesize that systolic and diastolic IVPG, a marker of normal left ventricular function, may be related to physiological asynchrony between basal and apical myocardial segments and that they can be attenuated, lost entirely, or even reversed when ventricular filling/emptying is impaired by acute regional ischemia or pressure overload, such as severe aortic stenosis.

### Objectives

#### Animal studies

1) Characterize IVPG along the cardiac cycle (systole and diastole);

2) Evaluate the effects of the ischemia and modulation by afterload;

3) Correlate the IVPG with myocardial segmental asynchrony, in basal, afterloaded and ischemic conditions.

#### Patient studies

1) Validate the invasive measurement of IVPG for systolic and diastolic function evaluation in patients with severe AS;

2) Apply this methodology to evaluate whether the IVPG improve in AS patients immediately after AVR;

3) Establish if IVPG changes correlate with the reduction in LV obstruction and improvement in LV function;

4) Correlate catheter measurements with preoperative echocardiography;

5) Evaluate the potential clinical applicability of the concepts derived from the experimental studies.

## Methods and Design

### Animal studies

The investigation conforms to the Guide for the Care and Use of Laboratory Animals published by the US National Institutes of Health (NIH Publication No. 85-23, Revised 1996).

#### Animal preparation

Male New Zealand White rabbits (Oryctolagus cuniculus, n = 6) are premedicated with ketamine hydrochloride (50 mg/kg im) and xylazine hydrochloride (5 mg/kg im). A femoral vein is cannulated, and a solution containing 20 meq KCl and 40 meq NaHCO_3 _in 500 ml of 0.9% NaCl is administrated at a rate of 8 ml·kg^-1^·h^-1 ^to compensate for perioperative fluid losses. A tracheostomy is performed, and mechanical ventilation is initiated (Harvard Small Animal Ventilator, model 683), delivering oxygen-enriched air. Respiratory rate and tidal volume are adjusted to keep arterial blood gases and pH within physiological limits. Anesthesia is maintained with a perfusion of midazolam (0.07 mg·kg^-1^·h^-1^), fentanil (0.003 mg·kg^-1^·h^-1^) and vecuronium (0.1 mg·kg^-1^·h^-1^). A 20-gauge catheter is inserted in the right femoral artery and connected to a pressure transducer to monitor heart rate and arterial pressure and to obtain samples for blood gas analysis. The heart is exposed by a median sternotomy, and the pericardium is widely opened. One silk suture is placed around the ascending aorta and then passed through a plastic tube to perform transient aortic occlusions during the experimental protocol. A limb electrocardiogram (DII) is recorded throughout.

#### Pressure measurements

Two 3-F high-fidelity micromanometer (SPR-524, Millar Instruments, Houston, Tex., USA) are inserted through an apical puncture wound into the LV cavity. One is pulled carefully back toward the endocardium and secured in place with a purse-string suture to measure apical LVP. The other is introduced until we can see the impact from the aortic valve on the pressure trace. The catheter then is pulled back 5 mm below the aortic valve so that it is located in the LV outflow-tract to measure basal LVP. The pressure transducers are calibrated against a mercury column and zeroed after stabilization for 30 min in a water bath at body temperature. The zero is set at the level of the right atrium. Recordings are made with respiration suspended at end expiration. Parameters are converted on-line to digital data with a sampling frequency of 1 kHz. LV pressures are measured at end-diastole (LVPED), at pressure nadir (LVP_min_) and at peak systole (LVP_max_). Peak rates of LV pressure rise (dP/dt_max_) and pressure fall (dP/dt_min_), as well as, time to dP/dt_min _are measured too. Relaxation rate are estimated with the time constant tau (τ) by fitting the isovolumetric pressure fall to a monoexponential function. We pretend to record continuously IVPG as apical minus outflow-tract LVP. Peak and average (area) IVPG are calculated during diastolic and systolic phases of cardiac cycle.

#### Sonomicrometry

Regional ventricular function is measured with two pairs of ultrasonic segment length gauges implanted in the circumferential direction of apical and basal left ventricular anterior midwall and connected to a sonomicrometer amplifier system (Triton Technology, San Diego, CA). At the end of the experiment, the animals are sacrificed with an overdose of anesthetics, and the position of the crystals and micromanometers are verified at necropsy. Segment lengths are measured at the end diastole (ED Length), at dP/dtmax and at mitral valve opening (MVO). Minimum segment length (Lengthmin) was measured as the minimum length preceding or coinciding with peak -dP/dt. Fractional shortening was calculated as the percent segment length change from end diastole to dP/dtmin at the outflow tract.

#### Experimental protocol

After complete instrumentation, we allow the animal preparation to stabilize for 30 min before the beginning of the experimental protocol. This consists in measuring apex to outflow-tract pressure gradient and apical and basal myocardial segments lengthening changes at basal, afterloaded and ischemic conditions.

#### Afterload manipulation

Sudden afterload elevations are performed by abruptly narrowing or occluding the ascending aorta during the diastole, as previously described [34,35]. In summary, this is achieved by pushing the plastic tube against the aorta with one hand while pulling the silk suture with the other hand. The analyzed intervention, therefore, is a selective alteration of afterload without changes of preload or long-term load history. The aortic clamp is quickly released to avoid neurohumoral reflex changes in cardiac function. The animal is stabilized for several beats before another intervention is performed.

#### Myocardial ischemia induction

Myocardial ischemia is induced by left coronary artery (LCA) ligation, after the first diagonal branch. Visible collateral arteries are tied as well to induce an antero-apical ischemia. Recordings are performed after 30 min. Mortality is documented.

### Patient studies

Full ethical approval for this study has been obtained from Ethics Committee of Centro Hospitalar de Vila Nova de Gaia, EPE. It is conducted in accordance with the principles of The Declaration of Helsinki, with the Portuguese laws and rules and subscribes to the principles outlined in the International Conference on Harmonisation of Good Clinical Practice [36].

All patients receive full explanation of study objectives, the operations to be performed, its risks and benefits and signed the informed consent form.

Any death or major complication during the study period requires the hospital ethical commission to be informed.

#### Study population

Patients between 65-80 years old (n = 12), both genders, with severe aortic stenosis (aortic valve area [AVA] < 1.0 cm^2^) referred for aortic valve replacement (AVR) are selected as eligible patients. Patients with any one of the following are excluded from the study: concomitant severe mitral regurgitation; mitral stenosis, regardless of severity; any prosthetic heart valve; coronary artery disease; concomitant aortic regurgitation; a history of surgical or percutaneous aortic valvuloplasty; history of ethanol abuse; and chronic obstructive pulmonary disease that are worse than mild as assessed clinically and/or confirmed by pulmonary function testing. (Table 1) The baseline and follow-up clinical variables and pharmacological data are obtained from a review of the medical records.

**Table 1:** Patient Eligibility Criteria

Inclusion	Exclusion
Symptomatic aortic valve stenosis	Concomitant > mild mitral regurgitation
Aortic valve area < 1.0 cm^2^	Mitral stenosis regardless of severity
First time cardiac surgery	Concomitant aortic regurgitation
Age 65-80 years old	Angiographic coronary artery disease
Signed informed consent	Chronic atrial fibrillation
	History of percutaneous aortic valvuloplasty
	History of ethanol abuse
	Chronic obstructive pulmonary disease > mild
	Urgent or emergent surgery
	Associated surgical procedure
	Creatinin > 1.5 ULN
	Inability to give informed consent

#### Intraoperative procedure

All patients undergo routine induction of general anesthesia, median sternotomy, and pericardiotomy. After great vessel cannulation and systemic heparinization, a high-fidelity pressure-volume catheter (Cardiovascular Millar Mikro-Tip^®^) is positioned from a small ascending aorta stab incision across the aortic valve. The 2 pressure sensors are positioned in the LV cavity in apex and in outflow-tract (sub-aortic valve) position. Appropriate anatomic placement is confirmed through the use of transesophageal echocardiography and visualization of appropriate chamber-specific waveforms.

#### Hemodinamic measurements

For each patient, during suspended ventilation, recordings of intracardiac pressure-volumes are obtained before cardiopulmonary bypass (CPB) beginning. After adequate data collection, the catheter is removed and placed in warm saline, myocardial arrest with full CPB support is obtained, and each patient undergoes biological AVR. After completely weaned from CPB and volume infusions from the CPB circuit to obtain adequate hemodynamics by increasing preload, after rezeroing, the catheter is repositioned across the aortic valve, and multiple hemodynamic measurements are obtained in several intervals during different stages of physiological stabilization. To compared with pre-CPB measurements catheters are matched at same LV end-diastolic pressure. During this period of data collection, no patient should require vasopressor, inotropic, or external pacing support. After data collection, the catheter is removed, systemic heparinization is reversed, and the operative procedure is concluded in the conventional fashion.

#### Echocardiography

All patients will have standard two-dimensional echocardiographic examinations before and after AVR. LV ejection fraction is assessed visually by a trained echocardiographer and entered into a database at the time of the examination. Anatomic and Doppler examinations and measurements are performed according to the recommendations of the American Society of Echocardiography. The aortic valve area is calculated using the continuity equation utilizing flow velocities in the LV outflow tract and across the valve. The pulmonary artery systolic pressure is calculated from the tricuspid regurgitation velocity signal using the simplified Bernoulli equation and estimated right atrial pressure based on inferior vena caval size. Doppler flow data is acquired from the LV outflow tract region in pulsed wave mode and from the aortic valve in continuous wave mode in the 5-chamber view. Peak velocities, calculated with resident software at the time of imaging, is used to calculate pressure gradients according to the modified Bernoulli equations and valve orifice areas according to the continuity equation approach.

### Statistical analysis

All analyses are performed using SPSS statistical software (SPSS 17.0, Chicago, IL).

#### Animal studies

Group data are presented as means ± SE and are compared using two-way ANOVA. Student-Newman-Keuls test is selected to perform pairwise multiple comparisons when significant differences are detected.

#### Patient studies

A two-tailed paired t test is used to compare patients before and after AVR. Differences are considered statistically significant at P < 0.05.

## Discussion

We expect 1) to validate the application of our invasive method to characterize the IVPG along cardiac cycle in physiological and pathological conditions; 2) to find a correlation between AS severity, diastolic dysfunction and IVPG impairment; 3) to provide new insights into the mechanical adaptation of LV to chronic afterload elevation and its response to unloading after AVR; 4) to show if the degree of hypertrophy parallels the severity of overload and if the assessment of IVPG can identify subtle contractile dysfunction; and 5) to promote the use of IVPG in clinical practice as another index of diastolic function and ventricular filling.

## Abbreviations

LV: left ventricle; IVPG: intraventricular pressure gradients; AS: aortic stenosis; AVR: aortic valve replacement; LVPED: left ventricular pressure at end-diastole; LVP_min_: left ventricular pressure nadir; LVP_max_: left ventricular pressure at peak systole; dP/dt_max_: peak rates of left ventricular pressure rise; dP/dt_min_: peak rates of left ventricular pressure fall; τ: time constant tau; ED Length: segment length at the end diastole; MVO: mitral valve opening; Length_min_: minimum segment length; LCA: left coronary artery; AVA: aortic valve area; CPB: cardiopulmonary bypass.

## Declaration of competing interests

The authors declare that they have no competing interests.

## Authors' contributions

MG and MJA contributed equally to this work. MG, MJA, JCM and ALM conceived and designed the protocol. MG and ALM contributed to the draft and final version of the manuscript. ALM and LV supervised the research project. All authors have read and approved the final manuscript.
